# Fabrication of Sesame Sticks-like Silver Nanoparticles/Polystyrene Hybridnanotubes and Their Catalytic Effects

**DOI:** 10.1038/srep39502

**Published:** 2016-12-21

**Authors:** Fang Peng, Qi Wang, Rongjia Shi, Zeyi Wang, Xin You, Yuhong Liu, Fenghe Wang, Jay Gao, Chun Mao

**Affiliations:** 1 National and Local Joint Engineering Research Center of Biomedical Functional Materials, Jiangsu Key Laboratory of Biofunctional Materials, School of Chemistry and Materials Science, Nanjing Normal University, Nanjing, 210023, China; 2 School of Environment, Nanjing Normal University, Nanjing, 210023, China; 3 School of Environment, The University of Auckland, Private Bag 92019, Auckland, New Zealand

## Abstract

A novel and efficient catalyst is one of the goals in the material field, and the involvement of nanoscience and technology has brought new vigor to the development of catalyst. This research aimed to develop a simple two-step route to fabricate Fe_3_O_4_@PS/PDA-Ag hybridnanotubes with size-controllable and highly dispersed silver nanoparticles (NPs). First, Fe_3_O_4_@PS nanotubes of a sound mechanical property were prepared using polystyrene (PS)/toluene solution containing highly dispersed oleic acid modified Fe_3_O_4_ particles in a commercial AAO template. Next, the facile technique was used to form *in situ* silver NPs on the surface of magnetic PS (Fe_3_O_4_@PS) nanotubes through dopamine coating. The catalytic effects of the prepared Fe_3_O_4_@PS/PDA-Ag hybridnanotubes with highly dispersed AgNPs were characterized using a range of analytical methods, including transmission electron microscopy, thermogravimetric analysis, UV-Visible spectroscopy, and X-ray diffraction. It was found that such prepared Fe_3_O_4_@PS/PDA-Ag hybridnanotubes had a large specific surface area. They possessed excellent activities in catalyzing the reduction of 4-nitrophenol (4-NP) by NaBH_4_ in the aqueous phase. Furthermore, they were readily separated from fluid and retrieved by an external magnet. Their catalyst activity and recyclability demonstrated that this approach we proposed had the potential to become a new idea and route for catalytic platform.

In recent years, noble metallic nanoparticle catalysts have been extensively studied both experimentally and theoretically owing to their remarkable catalytic activity and selectivity. For instance, silver nanoparticles (AgNPs) have received considerable attention as a potentially useful catalyst^1^,^2^,^3^,^4^,^5^. So far numerous methods have been developed to prepare stable noble metal nanoparticles with a controllable shape and size. However, it is still a challenge to select and assemble individual nanoparticles to form composite catalysts subsequently^6^. It is well-known that the size of AgNPs plays a critical role in catalysis. Smaller AgNPs tend to show a higher catalytic activity owing to their much higher surface-to-volume ratio^7^,^8^. However, smaller AgNPs can also aggregate very easily to minimize their surface area due to their higher surface energy, resulting in a remarkable reduction in their catalytic activities^9^. One practical and effective method of resolving the problem of agglomeration and/or enhancing their catalytic activities is to add AgNPs into solid supports such as polymer, carbon, and metal oxides of various nanostructures (e.g., spheres, fibers, mesoporous silica, and microporous metal-organic framework). Such formed composite catalysts tend to be highly effective at preventing the aggregation of small-sized AgNPs without losing their catalytic activity^7^.

In the literature, several nanotubes have been studied for their catalytic activities, one of which is polymer nanotubes. So far very few polymer nanotubes have been used to support noble metal nanoparticles, in sharp contrast to the large number of substrates of a spherical morphology. As an exciting class of one-dimensional nanomaterials with a large specific surface area, polymer nanotubes have been assessed for their potential applications in a wide variety of fields such as optoelectronic nanodevices, chemical sensors, and drug delivery^6^,^10^. Recently, polymer nanotubes have become the preferred drug delivery nanovehicles not only because they offer an enhanced drug loading capacity and stability, but also because they exhibit a good potential for surface modification and provide excellent pharmacokinetic control^11^,^12^,^13^. However, it is still a challenge to fabricate organic nanotubes with a controllable film thickness and diameter using the facile technique^10^.

Polymer nanotubes can be prepared in several ways, one of which is the well-known template method that is simple and very effective^14^,^15^. The array-structured polystyrene (PS) nanotubes of a stable mechanical property can be produced by filtrating a solution or melting normal molecular weight PS into anodic aluminum oxide (AAO) templates of only 200 nm pores. AAO is usually considered as one of the most useful templates because it has homogeneous pores and can be removed easily, even though the structure of polymer nanotubes depends strongly on the concentration of PS solution^16^,^17^,^18^. Another method of achieving multifunctional polymer coatings is to simply dip-coat objects in an aqueous solution of dopamine^19^,^20^. Nevertheless, PS nanotubes have seldom been used as catalyst supports.

Apart from PS, dopamine is another chemical that has found its use as catalyst supports. Under weak alkaline aqueous conditions, dopamine can generally self-polymerize into polydopamine (PDA) in the presence of oxygen at room temperature, and spontaneously deposits a thin layer of adherent coating onto various material surfaces. These deposits have a characteristic similar to that of the adhesive foot proteins secreted by mussels^21^. More importantly, the PDA layer can be used as a versatile platform for secondary reactions to create a variety of ad-layers, such as self-assembled monolayers through deposition of long-chain molecular building blocks, metal films by electroless metallization, and bioinert and bioactive surfaces via grafting of macromolecules^19^,^22^. Monodisperse PS nanotubes have been used as template nanotubes, with an additional coating step by PDA through self-polymerization of dopamine in alkaline aqueous. Subsequently, silver precursor-[Ag(NH_3_)_2_]^+^ ions in aqueous solution are successfully *in situ* reduced to AgNPs by PDA coating, and they are deposited on the surface of the PS/PDA nanotubes^21^.

Compared to AgNPs, magnetic nanoparticles are especially useful in biotechnology and medicine as they can perform such tasks as targeted drug delivery, bimolecular labeling and separation reliably^23^,^24^. In order to make them disperse more easily in the PS/toluene solution, oleic acid modified Fe_3_O_4_ nanoparticles are usually added to it. Because of the hydrophobic nature, magnetic nanoparticles can be dispersed easily in the PS/toluene solution. However, there is no known and proved route in effectively preparing Fe_3_O_4_@PS/PDA-Ag tubular nanocomposites at present. The purpose of this study is to develop a simple procedure to synthesize Fe_3_O_4_@PS/PDA-Ag tubular nanocomposites with size-controllable and highly dispersed AgNPs, and to test their catalytic effects through various analyses.

## Results

### Synthesis of Fe_3_O_4_@PS/PDA-Ag hybridnanotubes

**Figure 1:**
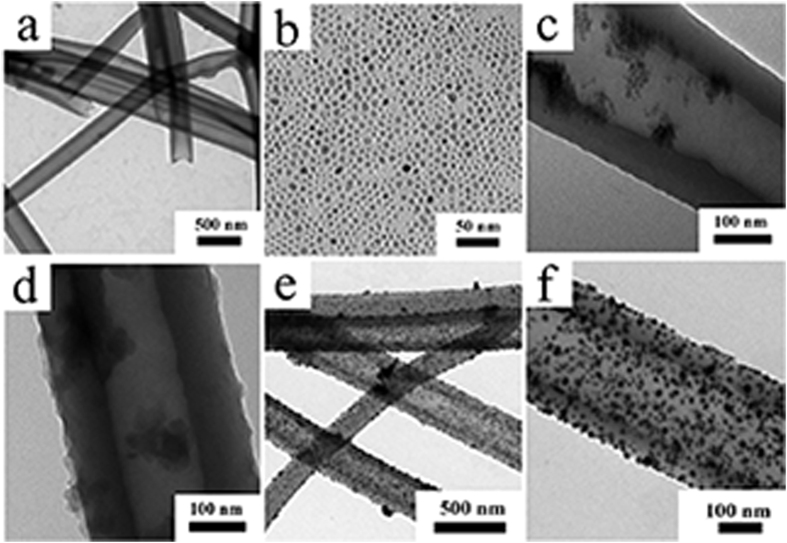
Transmission electron microscopy (TEM) images of PS nanotubes (**a**), Fe_3_O_4_ nanoparticles (**b**), Fe_3_O_4_@PS nanotube (**c**), Fe_3_O_4_@PS/PDA nanotube (**d**), Fe_3_O_4_@PS/PDA-Ag hybridnanotubes (**e** and **f**).

**Figure 2:**
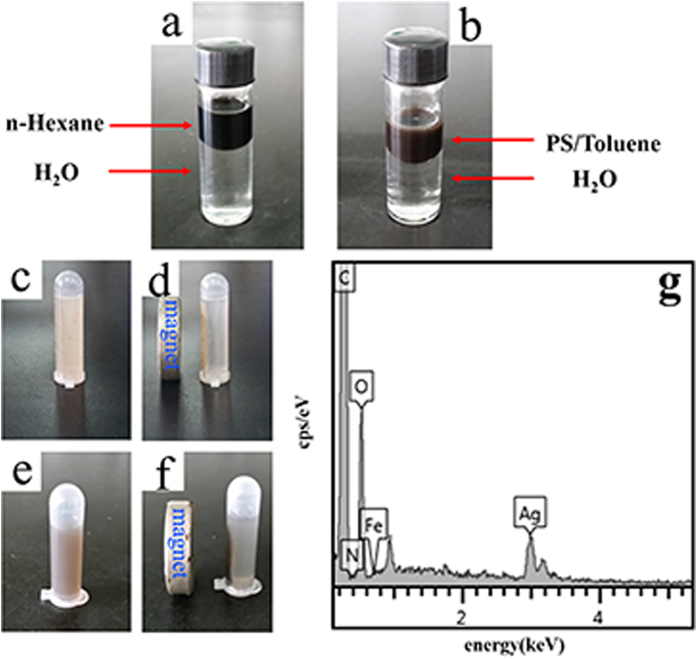
Acid modified Fe_3_O_4_ nanoparticles in n-hexane and water (**a**), oleic acid modified Fe_3_O_4_ nanoparticles in PS/toluene solution and water (**b**), Fe_3_O_4_@PS nanotubes separated from aqueous dispersion using an external magnet (**c, d**), Fe_3_O_4_@PS/PDA-Ag hybridnanotubes separated from aqueous dispersion using an external magnet (**e, f**), and the EDX spectrum of the Fe_3_O_4_@PS/PDA-Ag hybridnanotubes (**g**).

The PS nanotubes synthesized by wetting AAO template are considered a success as they are smooth and super-long (Fig. 1a). At a large scale they have a uniform size with an average diameter of 200 nm or so^9^,^21^. Their outer diameter is controlled by the diameter of the pores in the AAO template while their length is subject to the thickness of the template. In other words, it is possible to control their dimension. Since the inner diameter of nanotubes is not easily controllable, this inability can cause the thickness of the wall to vary. Despite this, the prepared nanotubes are still characterized by a uniformity in their dimension, and are flexible to adjust on command^13^. As revealed by TEM, the oleic acid modified Fe_3_O_4_ nanoparticles are about 5 nm in size (Fig. 1b). In contrast, the magnetic PS nanotubes have a hollow structure with a diameter of about 200 nm (Fig. 1c). If mixed with the PS nanotubes overnight at a pH of 8.5, dopamine is expected to be self-polymerized on the surface of the PS nanotubes^25^. The previously separated nanotubes were coated by PDA and nanotubes were turned much thicker (Fig. 1d). As shown in Fig. 1e and f, several round-shaped particles of about 10 nm in size are also present on the surface of the nanotubes. These particles were found to be Ag via the EDX analysis (Fig. 2f).

### Separation and characterization of Fe_3_O_4_@PS/PDA-Ag hybridnanotubes

The dispersed magnetic particles were entirely embedded or encapsulated in the walls of the PS nanotubes (Fig. 2a). The oleic acid modified Fe_3_O_4_ nanoparticles of a hydrophobic nature are well dispersed in hexane after water has been added. They are also well dispersed in the PS/toluene solution after the injection of water (Fig. 2b). Both the Fe_3_O_4_@PS nanotubes and Fe_3_O_4_@PS/PDA-Ag hybridnanotubes can be separated from aqueous dispersion using an external magnet (Fig. 2c,d,e,f). Owing to their strong magnetism, the Fe_3_O_4_@PS/PDA-Ag hybridnanotubes can be magnetically separated from aqueous solution within a few seconds, and redispersed evenly after demagnetization, rendering them economic and reusable in various applications^26^. The magnetic hysteresis loops obtained from the oleic acid modified Fe_3_O_4_ nanoparticles and the Fe_3_O_4_@PS/PDA-Ag hybridnanotubes are displayed in Supplementary Fig. S3. The saturation magnetization values of the oleic acid modified Fe_3_O_4_ nanoparticles and the Fe_3_O_4_@PS/PDA-Ag hybridnanotubes are 61.21 emu/g and 12.62 emu/g, respectively. The loss of magnetization is due to the presence of nanotubes. An increase in the nitrogen content in the EDX spectrum of the Fe_3_O_4_@PS/PDA-Ag hybridnanotubes yields further insights into polydopamine coating (Fig. 2g). After the Fe_3_O_4_@PS/PDA nanotubes were immersed in the AgNO_3_ solution, the reduction of Ag ions took place on their surface.

**Figure 3:**
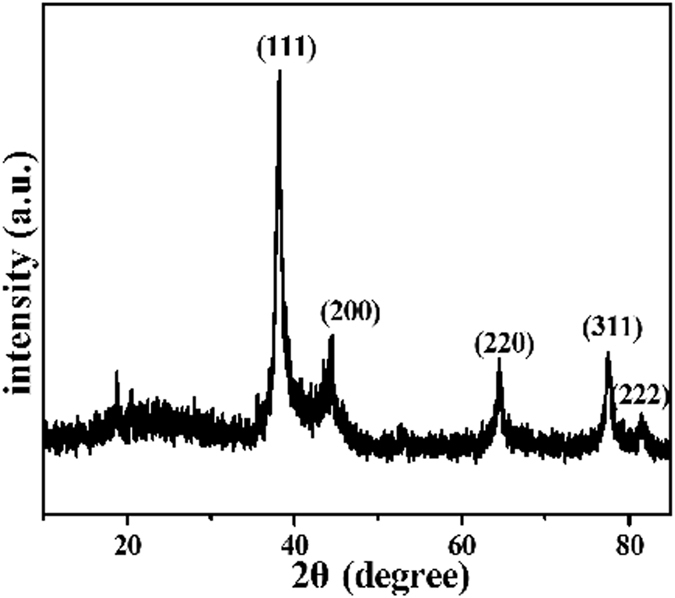
X-ray diffraction (XRD) patterns of the Fe_3_O_4_@PS/PDA-Ag hybridnanotubes.

The XRD pattern obtained from the oleic acid modified Fe_3_O_4_ nanoparticles has seven main peaks at 2θ of 30.20°, 35.08°, 43.20°, 53.82°, 56.98°, 62.06°, and 73.92°, corresponding respectively to the (220), (311), (400), (422), (511), (440), and (533) phases of the face-centered cubic (FCC) Fe_3_O_4_ crystal structure^23^ (Supplementary Fig. S4). The main peaks at 2θ = 38.24°, 44.28°, 64.46°, 77.42° and 81.56°correspond to the reflections of (111), (200), (220), (311), and (222) crystalline planes of the FCC crystal structure of Ag, respectively (Fig. 3). These findings are consistent with the ASTM standards (JCPDS Card No. 04–0783). They confirm the existence of the Ag nanoparticles in the outermost shell of the composite microspheres in the zero valent state^22^. In addition to the obvious Ag nanoparticles, the other part of the nanotube surface also shows the effect of Ag reaction in the EDX results. The characteristic structure of the 3-D nanotubes and the reduced PDA surface of Fe_3_O_4_@PS/PDA nanotubes present a solid evidence that silver ions can be reduced, deposited, and/or anchored through immersion in silver nitrate^27^. EDX characterization of the Fe_3_O_4_@PS/PDA-Ag hybridnanotubes show that the composite nanotubes were composed of C, O, N, Fe, and Ag, indicating the existence of AgNPs. These results suggest that the Fe_3_O_4_@PS/PDA-Ag hybridnanotubes are highly accessible with a sound ability to stabilize Ag nanoparticles^6^.

**Figure 4:**
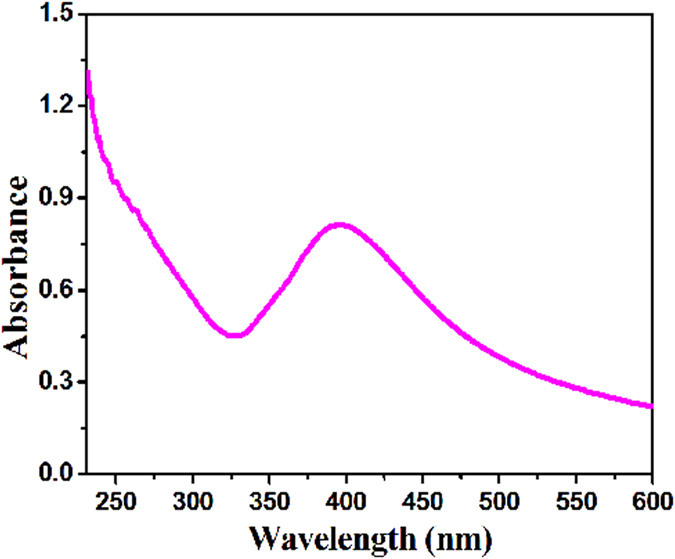
UV-vis absorbance spectrum of the Fe_3_O_4_@PS/PDA-Ag hybridnanotubes.

Shown in Fig. 4 is the UV-vis spectrum of the Fe_3_O_4_@PS/PDA-Ag hybridnanotubes. Their absorption spectrum exhibits an SPR band at around 416 nm due to the plasmon resonance, which confirms the presence of Ag nanoparticles in the Fe_3_O_4_@PS/PDA-Ag hybridnanotubes once again^6^.

**Figure 5:**
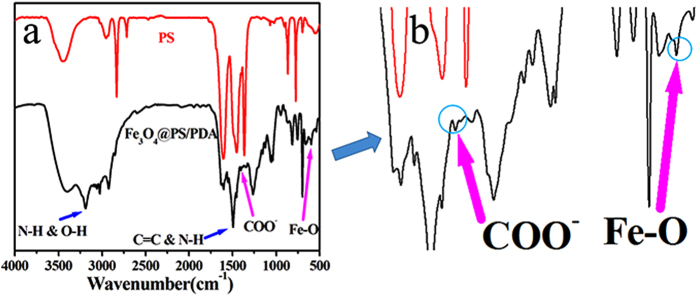
(**a**) FT-IR spectra of pure PS nanotubes and Fe_3_O_4_@PS/PDA nanotubes, (**b**) an amplified portion of FT-IR spectra.

Figure 5 illustrates the FT-IR spectra of pure nanotubes and Fe_3_O_4_@PS/PDA nanotubes. Their peak at 578 cm^−1^ corresponds to Fe-O vibration in Fe_3_O_4_. The oleic acid characteristic peaks at 2920 cm^−1^ (ν_as_-CH_2_), 2850 cm^−1^ (ν_s_-CH_2_), and 1420 cm^−1^ (ν_s_-COO^−^) confirm that oleic acid has modified the structure of the Fe_3_O_4_ nanoparticles^23^. After their surface has been covered with polydopamine, surface modification by dopamine solution results in the emergence of several new absorption signals. A broad absorbance between 3600 and 3100 cm^−1^ is ascribed to N-H/O-H stretching vibrations. The obvious peak at wavelength 1493 cm^−1^ in the spectrum of the PS-PDA nanotubes is attributed to C=C ring stretching and N-H from dopamine. These results demonstrate that the PDA composite layer has been incorporated into the surface of the PS nanotubes after dopamine modification^21^,^28^,^29^.

**Figure 6:**
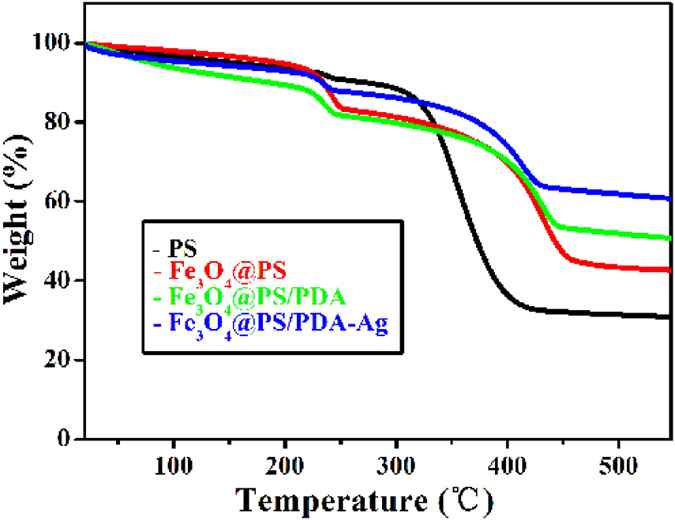
Thermogravimetric analysis (TGA) curves of four types of nanotubes.

The thermal stability of four types of nanotubes (PS, Fe_3_O_4_@PS, Fe_3_O_4_@PS/PDA, and Fe_3_O_4_@PS/PDA-Ag) is studied via TGA (Fig. 6). The pure PS nanotubes start to lose weight at around 300 °C, but the weight of residues is stabilized at about 30.8% of the total as temperature is elevated to 450 °C. In principle, the nanotubes should be converted to carbon at a high temperature under anaerobic circumstances. A comparison of the PS curve with the Fe_3_O_4_@PS curve shows that the content of encapsulated magnetic particles accounts for about 11.7% of the total. As temperature rose further to 450 °C, 8.2% of the residues were preserved owing to the PDA protective layer. On the other hand, the PDA layer facilitated deposition of silver nanoparticles, which, in turn, increased the proportion of residues from the organic and inorganic composite Fe_3_O_4_@PS/PDA nanotubes after sintering. The Fe_3_O_4_@PS/PDA-Ag curves in Fig. 6 clearly indicate that 10.0% of the silver nanoparticles were loaded^21^.

### Catalyzed performance of Fe_3_O_4_@PS/PDA-Ag hybridnanotubes

**Figure 7:**
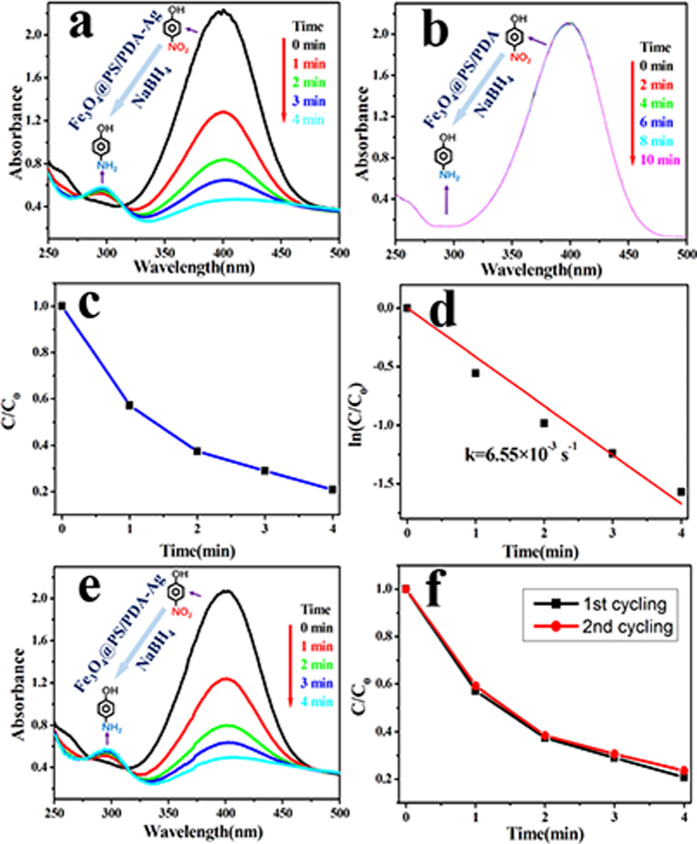
UV-vis absorbance spectra and the catalytic activity of nanotubes at different wavelengths and time. (**a**) Successive UV-vis absorbance spectra for the reduction of 4-NP by NaBH4 in the presence of Fe_3_O_4_@PS/PDA-Ag hybridnanotubes, measured at 1 min intervals; (**b**) Successive UV-vis absorbance spectra for the reduction of 4-NP by NaBH4 in the presence of Fe_3_O_4_@PS/PDA nanotubes, measured at 2 mins intervals; C/Co (**c**) and ln(C/Co) (**d**) ratio at different reaction time for the reduction of 4-NP catalyzed by the Fe_3_O_4_@PS/PDA-Ag hybridnanotubes; (**e**) Successive UV-vis absorbance spectra for the reduction of 4-NP by NaBH_4_ by the recovered Fe_3_O_4_@PS/PDA-Ag hybridnanotubes; (**f**) Catalytic activity of the Fe_3_O_4_@PS/PDA-Ag hybridnanotubes with double cycling uses.

The UV-vis spectrum of the 4-NP in water is used to study the catalyzed reduction in the experiments. In the absence of any catalysts both the absorbance value and the peak wavelength remain unchanged even for a couple of days. Addition of the Fe_3_O_4_@PS/PDA-Ag hybridnanotubes to the properly stirred mixture successively attenuates the intensity of peak absorbance of the nitro compound (Fig. 7a). This can also be appreciated from the discoloration of the characteristic yellow color of the solution. After the yellow color was completely discharged (i.e., at the end of the reaction), the peak caused by the nitro compound disappeared, as well. For the purpose of comparison, another experiment was undertaken with a mixture of 4-NP, the NaBH_4_ reducing agent, and the modified PS nanotubes containing no AgNPs. The results show almost no reduction in the absorbance of nitro compound at 400 nm in the UV-vis spectrum, suggesting no catalytic reduction of 4-NP (Fig. 7b). Furthermore, the color of the solution did not change with time at all. Moreover, there was no longer a 4-AP absorption peak at around 295 nm.

In order to explore the potential of the Fe_3_O_4_@PS/PDA-Ag hybridnanotubes as a strong catalyst, pseudo-first-order kinetics was used to evaluate the rate constant *k* for 4-NP reduction. The decomposition kinetics was understood according to physical chemistry principles. The results indicated that the preceding catalytic reduction reactions conform to the Langmuir-Hinshelwood apparent first-order kinetics model because of the superfluous NaBH_4_ used to prevent the 4-AP from aerial oxidation compared with 4-NP and catalyst. The rate constant *k* can be calculated from equation ln(C_t_/C_0_) = −kt, where *t* is the reaction time, *C*_*0*_ refers to the initial concentration of 4-NP, and C_t_ is the 4-NP concentration at time *t. k* was calculated to be 6.55 × 10^−3^ s^−1^ from the linear relationship in Fig. 7c and d. This value is superior to the reported figure for Ag nanoparticles of a smaller size as catalysts^5^,^30^. A contrast experiment of Ag nanoparticles used as catalyst with the same condition was performed by our research group (Supplementary Fig. S5). The UV-vis spectrum of the 4-NP in water was used to study the catalyzed reduction in the experiments. Results indicated that with the addition of Ag nanoparticles (0.1 mg/ml) to the reaction mixture caused the successive decrease in the intensity of the peak of the nitro compound. The peak caused by the nitro compound disappeared after 48 minutes. The rate constant *k* of Ag nanoparticles (0.1 mg/ml) was calculated to be 7.96 × 10^−4^ s^−1^, which much less than the rate constant *k* of the Fe_3_O_4_@PS/PDA-Ag hybridnanotubes we prepared.

Catalyst reusability is the main advantage of heterogeneous catalysts rather than homogeneous catalysts for industrial uses. A few studies have reported that the Ag catalyst could be recovered for further use in a consecutive cycle of catalysis^9^,^26^,^31^,^32^. In order to evaluate the reusability of the aforementioned nanotubes as catalysts, solid catalysts were recovered from the reaction mixture by an external magnet at the end of the first cycle of 4-NP reduction. As confirmed by the UV-vis spectrum in Fig. 7e, the recovered nanotubes are also highly active. The recovered nanotubes can be used as a catalyst in the second successive cycle of reduction reactions. According to Fig. 7f, there is little obvious catalytic loss in the second cycle, which is attributed to the high stability of AgNPs on the Fe_3_O_4_@PS/PDA-Ag hybridnanotubes. Therefore, the Fe_3_O_4_@PS/PDA-Ag hybridnanotubes have a very high catalytic activity, as well as a strong recyclability. Both features are conducive to industrial applications of the chemical^9^.

## Discussion

**Figure 8:**
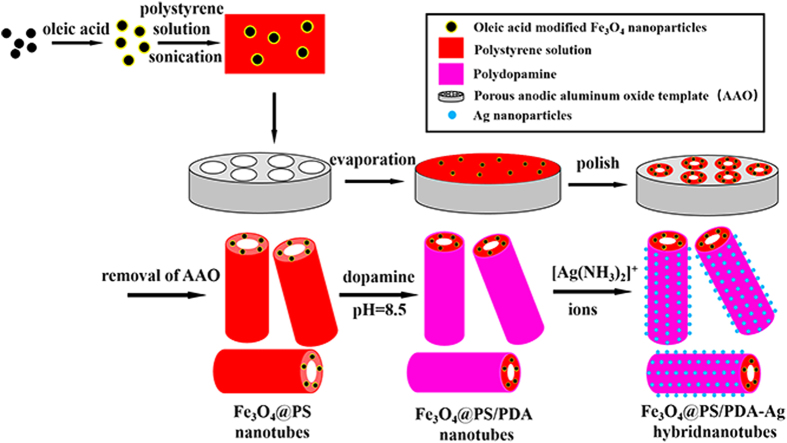
Mechanism and procedure of manufacturing the Fe_3_O_4_@PS/PDA-Ag hybridnanotubes.

In this study we developed an effective two-step method to synthesize Fe_3_O_4_@PS/PDA-Ag hybridnanotubes with size-controllable and highly dispersed silver NPs. The procedure of preparing Fe_3_O_4_@PS/PDA-Ag hybridnanotubes consisted of two steps. The first step was to prepare Fe_3_O_4_@PS nanotubes of a good mechanical property with PS/toluene solution containing highly dispersed oleic acid modified Fe_3_O_4_ particles in a commercial AAO template (200 nm pores) by a simple physical technique. In the experiments the oleic acid modified Fe_3_O_4_ nanoparticles with a hydrophobic nature were thoroughly dispersed in the PS solution^23^. Consequently, the magnetic nanoparticles were encapsulated or embedded in the PS nanotubes after the solvent had evaporated. The second step was to use the facile technique to form *in situ* silver nanoparticles on the surface of the Fe_3_O_4_@PS nanotubes through dopamine coating and immersing them in the silver nitrate solution^14^. In this process PDA acted as both the reduction agent and the template for the formation of AgNPs on the dopamine surfaces^26^. And [Ag(NH_3_)_2_]^+^ ions were first absorbed onto the surfaces of the Fe_3_O_4_@PS/PDA nanotubes because of the catechol and amine groups of the PDA shell. These adsorbed [Ag(NH_3_)_2_]^+^ ions on the surfaces of Fe_3_O_4_@PS/PDA nanotubes were then *in situ* reduced to metallic silver nanoparticles by the polydopamine coating. Finally, Fe_3_O_4_@PS/PDA-Ag hybridnanotubes were formed via the so-called electroless metallization procedure^21^. The entire process is illustrated in Fig. 8.

The catalytic effects of the prepared compounds were extensively tested and analyzed using UV-vis absorption spectra, SEM, XRD, TEM, FT-IR, and TGA. It is found that such prepared Fe_3_O_4_@PS/PDA-Ag hybridnanotubes with highly dispersed AgNPs have a diameter in the range of 10 nm. Their large specific surface area proved to have excellent activities in catalyzing the reduction of 4-NP by NaBH_4_ in the aqueous phase. In addition, the Fe_3_O_4_@PS/PDA-Ag hybridnanotubes can be easily recycled by an external magnet. These findings demonstrate that the Fe_3_O_4_@PS/PDA-Ag hybridnanotubes prepared through dopamine coating and surface modification offer a new platform for the preparation of an excellent catalyst using PS nanotubes-stabilized AgNPs.

## Methods

### Materials

AAO of 200 nm diameter pores was obtained from Whatman International Ltd. (U.K.). PS grains were obtained from Nanjing Yong Hong Chemical Co., Ltd. (Mw ~ 231.9 kg/mol, Mw/Mn = 1.98). Toluene (AR), ammonium hydroxide solution (NH_3_ ⋅ H_2_O) was purchased from Shanghai LingFeng Chemical Reagent Co. Ltd. Iron(II) chloride tetrahydrate (FeCl_2_ ⋅ 4H_2_O) was purchased from Shanghai Richjoint Chemical Reagent Co., Ltd. Oleic acid (OA) was purchased from Aladdin. Iron (III) chloride hexahydrate (FeCl_3_·6H_2_O), hexane, sodium hydroxide was purchased from Sinopharm Chemical Reagent Co., Ltd.

### Preparation of oleic acid modified Fe_3_O_4_ nanoparticles

The Fe_3_O_4_ nanoparticles were prepared by precipitation of Fe (III) and Fe (II) in an alkaline solution. A total of 13.5 g of FeCl_3_⋅6H_2_O and 6 g of FeCl_2_⋅4H_2_O were dissolved in 150 mL of distilled water under nitrogen at room temperature. Then 40 mL of NH_3_⋅H_2_O was added quickly to the solution under vigorous stirring to produce black precipitates. Six grams of oleic acid were added dropwise at a constant rate to the solution that had been heated at 80 °C for 1 hour. After an hour of reaction, the mixture was cooled to room temperature, and the oleic acid modified magnetic fluid was collected magnetically and washed using ethanol and hexane repeatedly. The black slurry was further dispersed in hexane for storage^23^.

### Preparation of magnetic PS nanotubes

The PS solution was prepared by dissolving 0.5 g PS grains in 10 ml toluene, and mixed with the oleic acid modified Fe_3_O_4_ nanoparticles (0.075 g) manually, followed by sonication for 30 minutes. A drop of the solution was placed on a microscope slide, on which a commercial AAO membrane was quickly placed. The solution entered the template pores completely along their inner wall in 2 seconds. Six hours later the solvent completely evaporated at room temperature. The AAO/magnetic PS composite membrane was removed from the microscope slide and polished by 1000 grit sand paper. Afterwards it was submerged in the 3 M sodium hydroxide solution to dissolve the template and to expose the Fe_3_O_4_@PS nanotubes^13^,^17^,^33^.

### Preparation of Fe_3_O_4_@PS/PDA-Ag hybridnanotubes

To coat magnetic PS nanotubes with the PDA shell, 5 mg of magnetic PS nanotubes and 20 mg of dopamine hydrochloride were dissolved in 10 mL of Tris buffer solution (10 mM, pH = 8.5). After shaking for 24 h at room temperature, the resultant products were separated by an external magnet and washed with ultrapure water and ethanol several times^26^. The typical strategy of manufacturing Fe_3_O_4_@PS/PDA-Ag hybridnanotubes starts with preparation of fresh [Ag(NH_3_)_2_]^+^ ion aqueous solutions at a concentration of 0.025 mol/L. Then ammonium hydroxide solution was added to the AgNO_3_ solution until it became transparent. Afterwards, 5 mg of Fe_3_O_4_@PS/PDA nanotubes were added to 10 mL of the freshly prepared [Ag(NH_3_)_2_]^+^ ion aqueous solution. The mixture was mechanically stirred at room temperature at a speed of 100 rpm for 1 h. The Fe_3_O_4_@PS/PDA-Ag hybridnanotubes were separated by an external magnet and washed with ultrapure water and ethanol several times. Finally, they were dried in a vacuum oven at room temperature for 24 h^21^.

### Characterization

All dried powder samples were gradually heated from 20 °C to 550 °C at a rate of 10 °C·min^−1^ under nitrogen atmosphere at a rate of 50 ml·min^−1^. UV-vis absorption spectra were measured at room temperature with a Cary 50 UV-vis spectrophotometer (UV-vis, VARIAN, U.S.A.) over the range of 200–600 nm. In addition, they were also characterized using a variety of analytical techniques, including scanning electron microscopy, transmission electron microscopy, X-ray diffraction, and transform infrared spectroscopy. The scanning electron microscopy (SEM, LEO1530VP, Germany) was equipped with an energy dispersive X-ray analysis (EDS). Transmission electron microscopy (TEM) (HITACHI H-7650, Japan) was designed to study their morphology. Anode rotating target X-ray diffraction (XRD, D/max 2500/PC, Japan) were carried out to identify crystal structure. The Fe_3_O_4_@PS/PDA-Ag hybridnanotubes powders deposited on a glass substrate were scanned at a rate of 0.02° (2θ) per second over the range of 10°–85° (2θ). The Fe_3_O_4_@PS/PDA-Ag hybridnanotubes were also analyzed using Transform Infrared Spectroscopy (FT-IR, Tensor 27, Bruker, Germany). Thermogravimetric analysis (TGA) was performed using the Perkin Elmer Instruments (TGA, Diamond TG/DTA, USA).

### Catalytic reduction experiments

Typically, an aqueous solution of NaBH_4_ (1.0 mL, 5.0 × 10^−2^ molL^−1^) was mixed with aqueous 4-NP solution (1.7 mL, 2.0 × 10^−4^ molL^−1^) in a quartz cell (1 cm path length). Subsequently, the above solution was mixed with Fe_3_O_4_@PS/PDA-Ag hybridnanotubes solution (0.3 mL, 1.0 mgmL^−1^) at 298 K. Then the catalytic process was monitored by measuring the changes with a UV−vis spectrophotometer.

## Additional Information

**How to cite this article**: Peng, F. *et al*. Fabrication of Sesame Sticks-like Silver Nanoparticles/Polystyrene Hybridnanotubes and Their Catalytic Effects. *Sci. Rep.* **6**, 39502; doi: 10.1038/srep39502 (2016).

**Publisher's note:** Springer Nature remains neutral with regard to jurisdictional claims in published maps and institutional affiliations.

## Supplementary Material

Supplementary Information
