# Supplementary material for: Fabrication of Sesame Sticks-like Silver Nanoparticles/Polystyrene Hybridnanotubes and Their Catalytic Effects
Source: Sci Rep. 2016 Dec 21;6:39502. doi: 10.1038/srep39502 (PMC5175140; doi:10.1038/srep39502)
Supplement: Supplementary Information [file srep39502-s1.pdf]

Fabrication of Sesame Sticks-like Silver Nanoparticles/Polystyrene Hybridnanotubes and Their Catalytic Effects

Fang Peng[1,+], Qi Wang[1,+], Rongjia Shi[1], Zeyi Wang[1], Xin You[1],Yuhong Liu[1], Fenghe Wang[2,*], Jay Gao[3], Chun Mao[1,*],

Supplementary information

The dispersion situations of AgNPs that prepared by different methods were studied by the transmission electron microscopy (TEM). The sodium citrate modified AgNPs (0.1mg/ml) that reduced by $NaBH_4$ had a good dispersion characteristic and uniform shape (*J. Phys. Chem. B, 109, 19208-19212 (2005)*) (Supplementary Fig. S1 a). However, while increasing concentration of sodium citrate modified AgNPs to 1 mg/ml, the apparent aggregation and sedimentation were observed (Supplementary Fig. S1 b). In contrast, when the concentration of Fe_3O_4@PS/PDA-Ag hybrid nanotubes we prepared was up to 10 mg/ml, the AgNPs were still stable that decorated PS nanotubes without aggregation (Fig. S1 c).

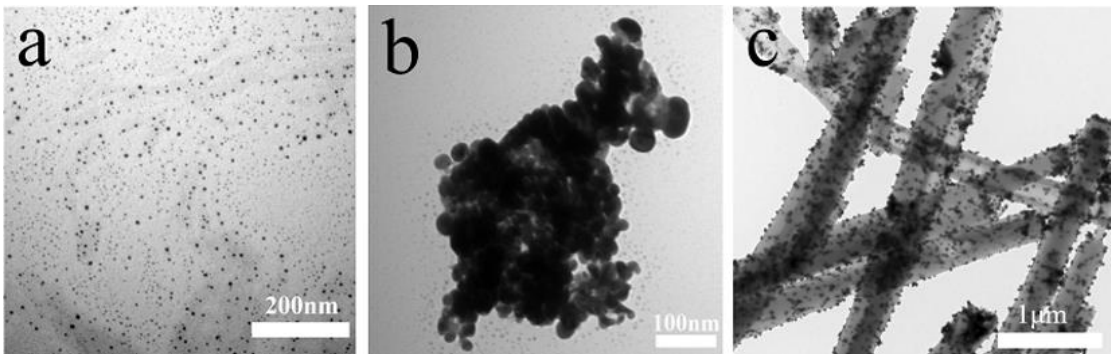

Supplementary Figure S1. Transmission electron microscopy (TEM) images of sodium citrate modified Ag nanoparticles (0.1 mg/ml) (a), TEM images of sodium citrate modified Ag nanoparticles (1 mg/ml) (b), TEM images of Fe_3O_4@PS/PDA-Ag hybridnanotubes (10 mg/ml) (c).

Polymer nanotubes can be prepared in several ways, one of which is the well-known template method that is simple and very effective. Guojun Song reported PS nanotubes were successfully obtained and the wall thickness of PS nanotubes prepared by the 2.5 wt%, 5.0 wt%, and 10.0 wt% PS solution were 50 nm, 70 nm, and 80 nm, respectively. The structure of polymer nanotubes depends strongly on the concentration of PS solution (*J. Mater. Chem. B 2, 1327-1334 (2014); J. Mater. Res. 19, 3324-3328 (2004)*). Further, the outer diameter of PS nanotube is easy controlled by the diameter of the pores in the AAO template while their length is subject to the thickness of the template. At present, it's easy to buy AAO template with a series of pore size from the market. So we think that the structure flexible to adjust on command. For example, we have prepared PS nanotube with the outer diameter of 100 nm by using a commercially available AAO template (Supplementary Fig. S2).

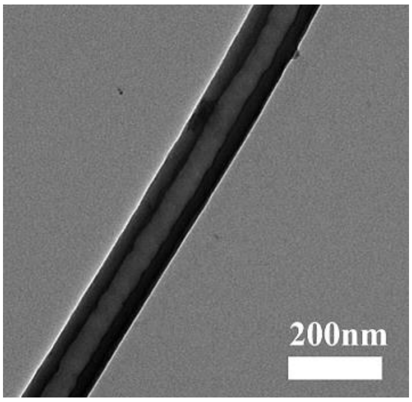

Supplementary Figure S2. PS nanotube with the outer diameter of 100 nm.

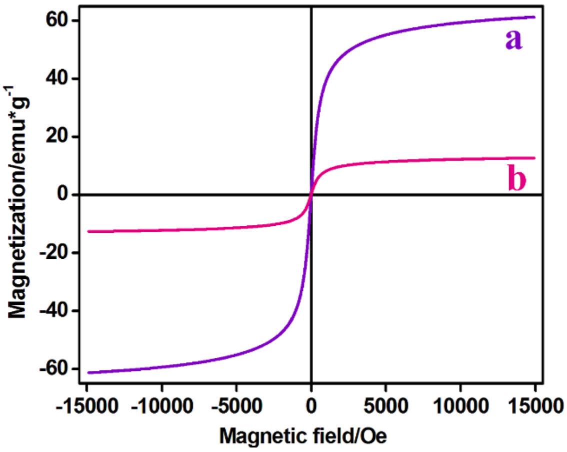

Supplementary Figure S3. Magnetization curves of (a) the oleic acid modified Fe_3O_4 nanoparticles and(b) the Fe_3O_4@PS/PDA-Ag hybridnanotubes.

As shown in Supplementary Fig. S4, the XRD pattern obtained from the oleic acid modified Fe_3O_4 nanoparticles has seven main peaks at 2θ of 30.20 °, 35.08 °, 43.20 °, 53.82 °, 56.98 °, 62.06 °, and 73.92 °, corresponding respectively to the (220), (311), (400), (422), (511), (440), and (533) phases of the face-centered cubic (FCC) Fe_3O_4 crystal structure, respectively (*Biomaterials 33, 3013-24 (2012)*).

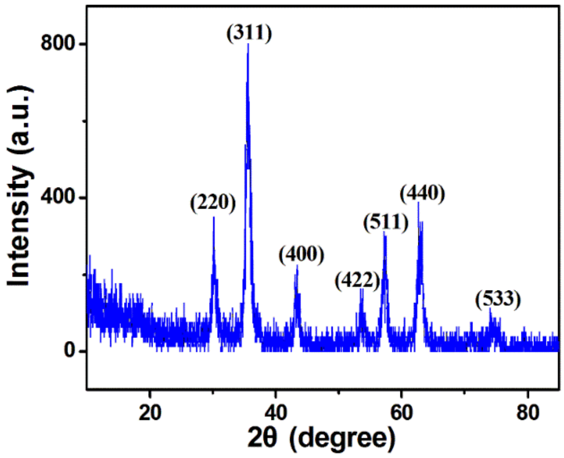

Supplementary Figure S4. X-ray diffraction (XRD) patterns of the oleic acid modified Fe_3O_4 nanoparticles.

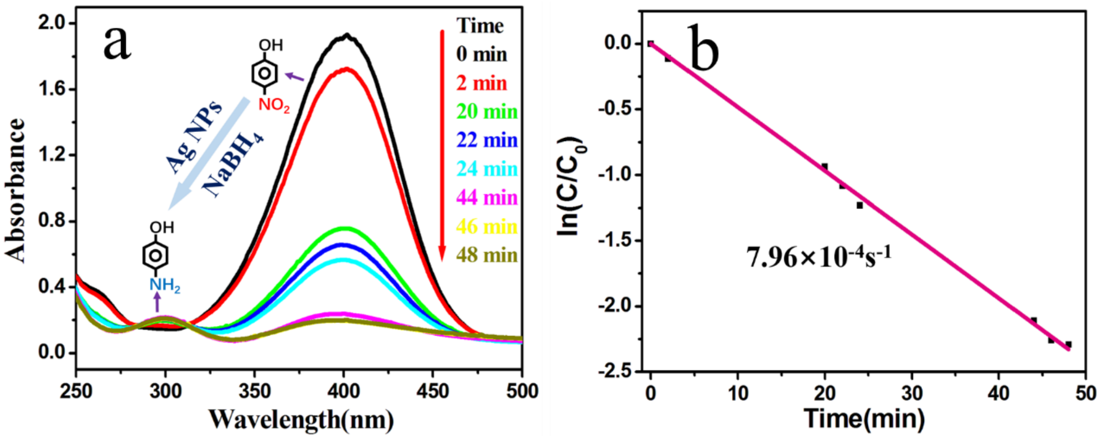

Supplementary Figure S5. (a) Successive UV-vis absorbance spectra for the reduction of 4-NP by $NaBH_4$ in the presence of Ag nanoparticles (0.1mg/ml), measured at 2 min intervals; (b) ratio at different reaction time for the reduction of 4-NP catalyzed by the Ag nanoparticles (0.1mg/ml).
